# O6-2 Physical activity policy actions in Europe: the MOVING benchmarking tools

**DOI:** 10.1093/eurpub/ckac094.042

**Published:** 2022-08-29

**Authors:** Kate Oldridge-Turner, Margarita Kokkorou, Ioana Vlad, Arnfinn Helleve, Anne-Siri Fismen, Jonas Rekdal Mathisen, Janetta Harbron, Gaironeesa Hendricks, Knut Inge Klepp, Kate Allen

**Affiliations:** 1 World Cancer Research Fund International, London, United Kingdom; 2 Norwegian Institute for Public Health, Oslo, Norway; 3 University of Cape Town, Cape Town, South Africa

**Keywords:** policy, physical activity, benchmarking tool

## Abstract

**Background:**

Global research shows a strong link between physical activity and risk of developing non-communicable diseases. To increase physical activity levels, governments must design and implement a comprehensive set of policy actions across a range of areas. To aid governments in this process, the MOVING benchmarking tool was developed to assess the strength of policies across three domains that make up a comprehensive approach to physical activity policy.

**Methods:**

The MOVING benchmarking tool, which was developed using a consultative process that reviewed evidence on physical activity policy design and existing benchmarking tools, built on the policy areas of the MOVING framework. It values the strength of policy design, based on evidence-informed and aspirational attributes of effective policy-making that impacts health-related behaviours, with a focus on adolescents. The tool was applied to a set of physical activity policies from five countries: Netherlands, Norway, Poland, Portugal and UK. These countries were selected based on their participation in the EU-funded CO-CREATE project, which focuses on prevention of overweight and obesity among adolescents. Relevant physical activity policies in each participating country were identified based on a comprehensive scan, with a set methodology.

**Results:**

The MOVING benchmarks assessed the strengths and weaknesses in the design of policies across the policy areas of the MOVING policy framework. They produced an overall assessment of policy environments for physical activity across the five countries, as well as an assessment of each individual policy area within the framework, such as walking and cycling infrastructure and active transport. Further, by allowing a fast assessment of many physical activity policies, the benchmarking tool enabled an analysis of the interplay of single policies and drawing conclusions about the overall policy environment in the five countries.

**Conclusion:**

The MOVING benchmarking tools can identify gaps and assess the strength of actions taken by national governments to promote physical activity, and can be used by policymakers, researchers and civil society to inform advocacy for and design of policies. The scores generated by the benchmarking tool will be amalgamated into an overall policy index for 27 European countries.

